# Adnexal Torsion: A Retrospective Analysis From a Tertiary Care Teaching Hospital in Northern India

**DOI:** 10.7759/cureus.17792

**Published:** 2021-09-07

**Authors:** Menka Verma, Vandana Bhuria, Meenakshi Chauhan, Smiti Nanda, Pushpa Dahiya, Savita R Singhal

**Affiliations:** 1 Obstetrics and Gynaecology, Pt. B. D. Sharma Post Graduate Institute of Medical Sciences (PGIMS) Rohtak, Rohtak, IND

**Keywords:** adnexa, laparotomy, ovarian, reproductive, torsion

## Abstract

Background

Adnexal torsion is an acute gynecological emergency presenting with acute abdomen which can be missed owing to non-specific symptoms. Among reproductive-age women, conservative surgery is preferred. The present study was a retrospective analysis of adnexal torsion cases reported to a tertiary care teaching hospital in Northern India. The purpose of the study was to describe the demography, clinical features, diagnostic and treatment modalities, and prognosis of adnexal torsion cases.

Methods

Surgically proven adnexal torsion case records were retrieved and data were entered in an excel sheet from a period of two and half years from January 2018 to June 2020.

Results

There were 28 cases with an age range of 7-85 years (median age 24 years) with lower abdominal pain and nausea/vomiting symptoms. The majority were in the reproductive age group (71.4%). A Colour Doppler was done which detected 75% (12/16) of the ovarian torsion cases. The size of the adnexal torsion was 5-10 cm in 60.7% with right-sided torsion seen in 57.14%. Detorsion and salpingo-oophorectomy was done in 14 (50%) and 11 (39.2%) cases, respectively. Histopathological examination revealed hemorrhagic/necrotic infarcts (54.2%) and dermoid cysts (33.3%).

Conclusions

Owing to non-specific symptoms, adnexal torsion is diagnosed with strong clinical suspicion as routine ultrasonography diagnosed only 7.1% in the present study. Conservative surgery is preferred in the reproductive age group.

## Introduction

Adnexal torsion is one of the few acute surgical emergencies of gynecological origin. Ovarian torsion is defined as a partial or complete rotation of the ovarian vascular pedicle causing obstruction to venous outflow and arterial inflow [1,2]. Adnexal torsion involves twisting of the fallopian tube along with the ovarian vascular pedicle [3]. About 3% of cases presenting with acute abdominal discomfort in the emergency room are due to adnexal torsion. The majority of these instances occur in women of reproductive age and are frequently associated with a cyst or tumor, the most common of which is mature cystic teratoma [1,4,5]. Because of nonspecific symptoms, there can be delays in diagnosing adnexal torsion. Imaging using ultrasound with Doppler flow, CT scan, and MRI helps in diagnosis. Ultrasound is the most common initial approach for diagnosis of adnexal mass with Doppler flow to rule out torsion. Laparoscopy is the preferred procedure for diagnosis and treatment of adnexal torsion and conservative surgery like detorsion with cystectomy or cyst aspiration or oophoropexy is preferred rather than removal of the adnexa whenever possible.

## Materials and methods

The present study was conceived to describe the demography, clinical features, treatment, and prognostic factors of adnexal torsion. The index study was conducted in a tertiary care teaching institute of North India with a yearly delivery load of 8000 to 10,000 and is a referral center. The institute is an established one with more than 50 years and attracts patients from adjoining states. There are a total of 26 consultants of obstetrics and gynecology (out of which eight are senior consultants with experience of more than 20 years) who are directly or indirectly involved in the care of patients. A retrospective record analysis of inpatient surgical registry from January 2018 to June 2020 identified 28 women with surgically proven ovarian torsion. Clinical information obtained from the patient’s medical records included demographic profile, obstetric and menstrual history, clinical presentation and laboratory investigations. Imaging [ultrasonography (USG) with radiological findings, CT scan, and MRI imaging], operative findings, and histopathological reports were also noted. Ovarian torsion is defined as the twisting of an ovary around its ligamentous support which can result in compromised vascularity. Adnexal torsion is a term that includes either the ovary, fallopian tube, or both [2]. All cases with proven adnexal torsion were retrieved and reviewed by the lead researcher and the data used for analysis were extracted directly from case reports. The present study was approved by the Institutional Ethics Committee of Pt. B. D. Sharma Post Graduate Institute of Medical Sciences (Approval no. - BREC/21/89).

Statistical methods

The data were entered in a Microsoft Excel spreadsheet (Microsoft® Corp., Redmond, WA). Continuous variables are presented as means ± standard deviation whereas categorical parameters are presented in frequency and percentage.

## Results

Twenty-eight surgically proven adnexal torsion patients were included in the study. The records of the confirmed cases were found complete with only symptomatic treatment given at the periphery level before referring to the tertiary care center. The median age of women in our study was 24 years (7-85 years). There were three (10.71%) patients in the premenarchal age group, 20 women (71.42%) in the reproductive age group, and five (17.85%) in the postmenopausal group. Out of 28, 14 women were unmarried and two (7.14%) patients were antenatal; of these one was in the first and the other was in the second trimester (Table 1).

**Table 1:** Demographic profile of study subjects

Variable	Category	Frequency (Percentage)
Age (in years)	5-12 years	3 (10.7%)
	13-40 years	20 (71.4%)
	>40 years	5 (17.9%)
Married	Yes	14 (50%)
	No	14 (50%)
Pregnancy	Yes	2 (7.1%)
	No	26 (92.9%)
Menopause	Yes	5 (17.9%)
	No	23 (82.1%)

Lower abdominal pain was the commonest symptom present in all the patients (100%) associated with tenderness. The associated symptoms were nausea and vomiting in 21 (75%), fever in five (17.85%), altered bowel habit in two (7.14%) and dysuria in one (3.57%) woman. Abdominal examination revealed a palpable mass in 14 (50%) women. Laboratory investigations revealed leucocytosis in two (7.14%) women. Ultrasound was performed in all women whereas Colour Doppler and MRI scan were done in 16 and three patients, respectively. Colour Doppler diagnosed 75% of the torsion cases which was higher as compared to other modalities (ultrasonography, USG and magnetic resonance imaging, MRI). MRI was indicated to evaluate the signs of complications and to arrive at a more definitive diagnosis. Ovarian cyst was reported in 18 cases (64.28%) in ultrasound (Table 2).

**Table 2:** Proportion of women detected as ovarian torsion with different imaging techniques USG: Ultrasonography

	Number of women having imaging	Number of women with detected ovarian torsion
USG	28 (100%)	2 (7.1%)
Colour Doppler done	16 (57.14%)	12 (75%)
MRI done	3 (10.7%)	2 (66.7%)

All the cases in our study were managed surgically with laparotomy. However, the timing of surgery varied due to delayed reporting to the hospital. Out of 28 women, laparotomy for ovarian torsion was done within 24 hours only in 14 patients, and in the rest of the women, surgery could be done after 24 hours of the onset of symptom. There was the presence of hemorrhage and infarcts in 13 patients who presented late with no difference in results. The operative findings during surgery were also recorded which included adnexal structures involved, site, size, and a number of twists. The operative findings were isolated torsion ovary in 12 cases (42.85%), combined ovarian and fallopian tube in 14 (50%), and para-ovarian cyst in two (7.14%). The present study identified tumors, bulky ovaries, and adhesions as risk factors for adnexal torsion.

**Figure 1:**
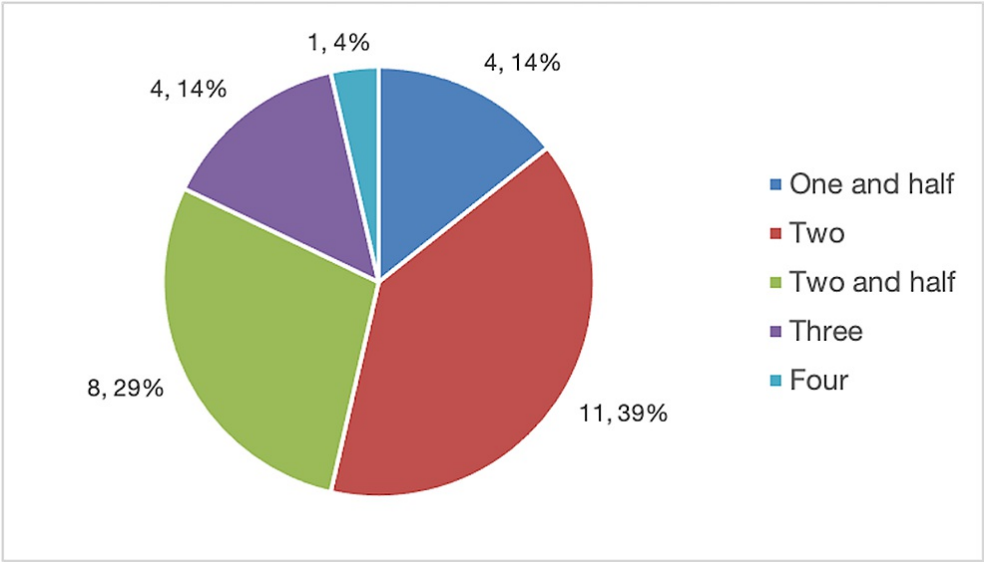
Distribution of patients showing the twists observed during surgical management

The size of the mass in adnexal torsion was <5 cm in four cases (14.28%), 5-10 cm in 17 cases (60.71%), and >10 cm in seven cases (25%). Right-sided torsion was seen in 16 cases (57.14%), left-sided in 11 cases (39.28%), and bilateral in one case (3.57%), who was a postmenopausal woman with an adnexal mass on both ovaries. The number of twists was also noted with median twists 2 (1.5-4.0) (Figure 1).

Detorsion with ovarian fixation was done in four cases (14.28%), detorsion with cystectomy in 10 (35.71%), salpingo-oophorectomy in 11 cases (39.28%) and abdominal hysterectomy with bilateral salpingo-oophorectomy (BSO) in three cases (10.71%).

Histopathological examination was done for 24 specimens. Eight women (33.3%) had a dermoid cyst, 13 (54.2%) had hemorrhagic and necrotic infarcts, one each (4.2%) had mucinous cystadenocarcinoma (malignant), mucinous cyst fibroma, and simple cyst, respectively. None of the menopausal surgical torsion cases were using hormone replacement therapy (HRT).

## Discussion

Adnexal torsion can occur in all age groups but is most commonly seen in reproductive age groups [4,5]. In our study also, the majority of the women (71.4%) were of reproductive age. The median age of women with ovarian torsion in the present study was 24 years which is comparable to the study by Gupta et al. (26 years) [6]. The most common pathologic conditions are mature cystic teratomas, followed by corpus luteum and para-tubal cysts, respectively. It occurs predominantly in the first trimester with only 5% occurring after 20 weeks of gestation, as the free space in the pelvis decreased [7-9]. In our study, there were two women who presented in early pregnancy with pain in abdomen and were diagnosed with ovarian torsion. On the contrary, a study by Resapu et al. reported a 36% incidence of ovarian torsion in pregnancy [10]. The incidence of torsion with pregnancy was low and this is in accordance with the other studies [11]. This finding, however, needs to be confirmed with studies with a large sample size. The rate of adnexal torsion during pregnancy varies from 1% to 14% and is more common with masses between 6 and 8 cm [12].

Symptoms in women with adnexal torsion are usually nonspecific that leads to delay in the diagnosis. A high level of clinical suspicion of adnexal torsion along with radiological imaging helps in early diagnosis and timely management. Lower abdominal pain was the main symptom in all 28 patients. Houry and Abbott reported in their 15-year review of 87 surgically confirmed ovarian torsion where stabbing pain (90%), nausea and vomiting (70%), and mild tenderness on abdominal examination (35%) were common symptoms [12]. A similar profile of symptoms was described by Balci et al. where pelvic pain and nausea/vomiting were predominant symptoms [13].

Ultrasound examination has a sensitivity of 40-75% to diagnose adnexal torsion [14]. In our study, ultrasound was performed in all patients and Colour Doppler was done in 16 patients which revealed 12 (75%) of the ovarian torsion cases. Colour Doppler demonstrates the presence of flow of blood. During ovarian torsion, venous flow is obliterated first followed by arterial flow [15]. Ovarian cyst was reported in 18 cases (64.28%) in ultrasound. Grunau et al. in their study of 55 proven ovarian torsion cases reported a significant presence of ovarian cysts [16]. Similar results were reported by White and Stella with 51.9% ovarian cysts [17]. Twisted adnexal masses are often found in the midline and anterior to the uterus.

Oltmann et al. and Budhram et al. reported that there is more likelihood of torsion when the size of the ovarian mass is 5 cm or larger [18, 19]. In the present study, there were 17 cases (60.71%) having 5-10 cm size of adnexal mass which had undergone torsion. This finding suggests a higher adnexal mass (>5 cm in size) as a risk factor for adnexal torsion. The mean size of the torsed ovary was 9.5 cm (range 1-30 cm) with 89% of the ovary had a size greater than 5 cm as reported by Houry and Abbott [12].

In our study, right-sided torsion was seen in 16 cases (57.14%), left-sided in 11 cases (39.28%), and bilateral in one case (3.57%), who was a postmenopausal woman with an adnexal mass on both ovaries. A similar predominance of right-side torsion was reported in a study from Pondicherry, India [6]. Torsion of the right ovary was more common than the left, probably due to the presence of a sigmoid on the left side and a longer right ligament of the ovary [20]. The current study had the presence of twists with a median value of 2.0 which coincided with the finding of a retrospective study from Southern India involving 81 cases [6].

All the patients underwent laparotomy as the facilities for laparoscopy do not exist in the emergency department at our hospital. Detorsion with ovarian fixation was done in four cases (14.28%), detorsion with cystectomy in 10 (35.71%), salpingo-oophorectomy in 11 cases (39.28%), and abdominal hysterectomy with BSO in three cases (10.71%). Similar results were also reported by a retrospective study involving 81 cases where 30 (37.0%) underwent detorsion with cystectomy [6]. Another study done in Turkey had 10.6% of cases who underwent total abdominal hysterectomy-bilateral salpingo-oophorectomy (TAH-BSO) under laparotomy [13].

Regarding pathological findings, the commonest presentation in the current study was dermoid cyst (33.3%) whereas Balci et al. reported teratoma (32.0%) and follicular cyst (14.6%) as the commonest [13]. Extensive necrosis/infarction (78.3%) was reported by Gupta et al. among ovarian torsion cases who underwent radical surgery [6].

The study, however, had certain limitations. The study has a small sample size which could have been increased to have more external validity. As this was a retrospective study, bias due to incomplete data might have crept in. Also, cases with the less typical presentation of ovarian torsion who did not require surgery may have been missed.

## Conclusions

Diagnosis of adnexal torsion is not easy because of non-specific symptoms so it requires good clinical awareness. Strong clinical suspicion is the key factor to diagnose adnexal torsion. Ultrasound with Doppler helps in diagnosing adnexal mass with torsion, so it should be done whenever one suspects a torsion. Conservative surgery is the preferred mode of treatment for patients in the reproductive age group.
